# Supplementary material for: Genetic diversity of Collaborative Cross mice implicates FFAR3 as a target for ILC2 anti-inflammatory reprogramming
Source: Nat Commun. 2026 Jan 3;17:1053. doi: 10.1038/s41467-025-67813-2 (PMC12847941; doi:10.1038/s41467-025-67813-2)
Supplement: Supplementary file 1 — Supplementary Information [file 41467_2025_67813_MOESM1_ESM.pdf]

Supplementary Figure 1

A.

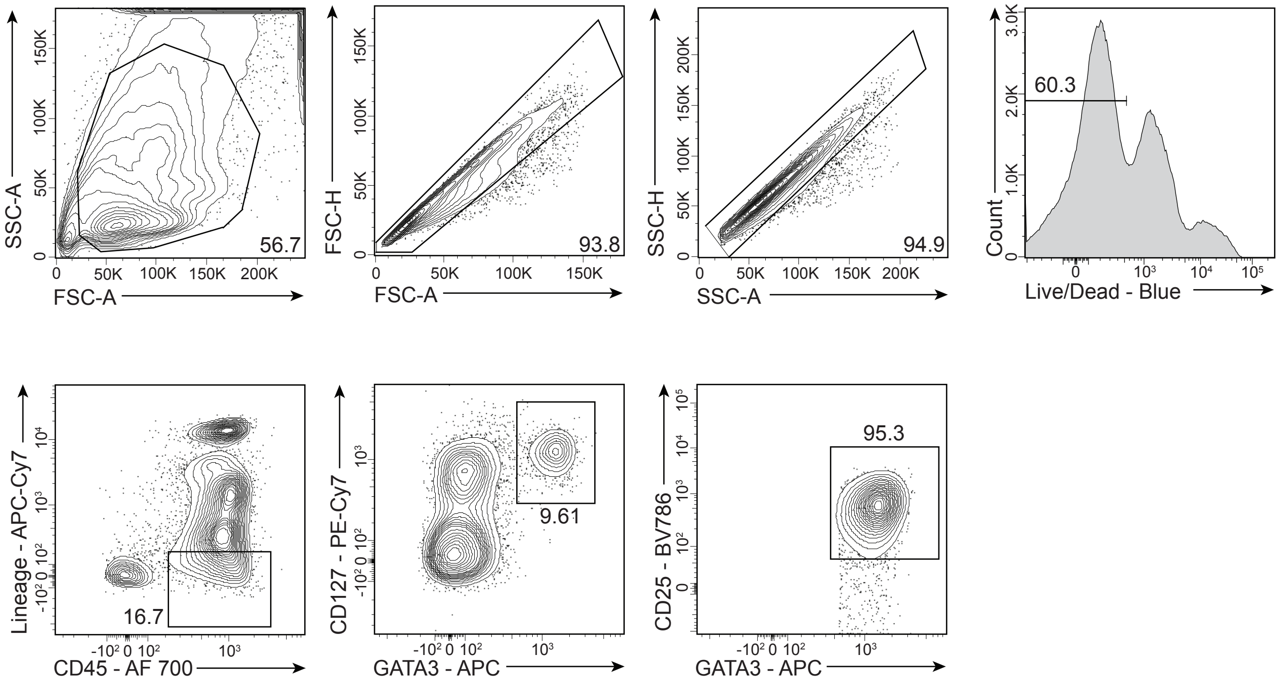

Supplementary Figure 1.

Flow cytometry gating strategy for quantifying ILC2s in different CC mouse strains. (A) ILC2s were quantified for Figure 1 with the provided gating strategy, read from left to right. Cells from each strain were isolated from the 4 lobes of the right lung, according to the protocol outlined in the methods section. ILC2s in Figure 1 are represented as a percentage of live lung cells. This percentage is calculated from the number of ILC2s, defined as live singlet cells (FSC/SSC) that are Lineage-, CD45+, CD127+, GATA3+, and CD25+, as a proportion of live singlet cells (top right plot). This ratio is multiplied by 100 to attain a percentage. Lineage staining includes a cocktail of monoclonal antibodies against CD3, CD4, CD5, CD11b, CD45R, Ly-6B.2, Gr-1, Ter-119, and FCεRI.

Supplementary Figure 2

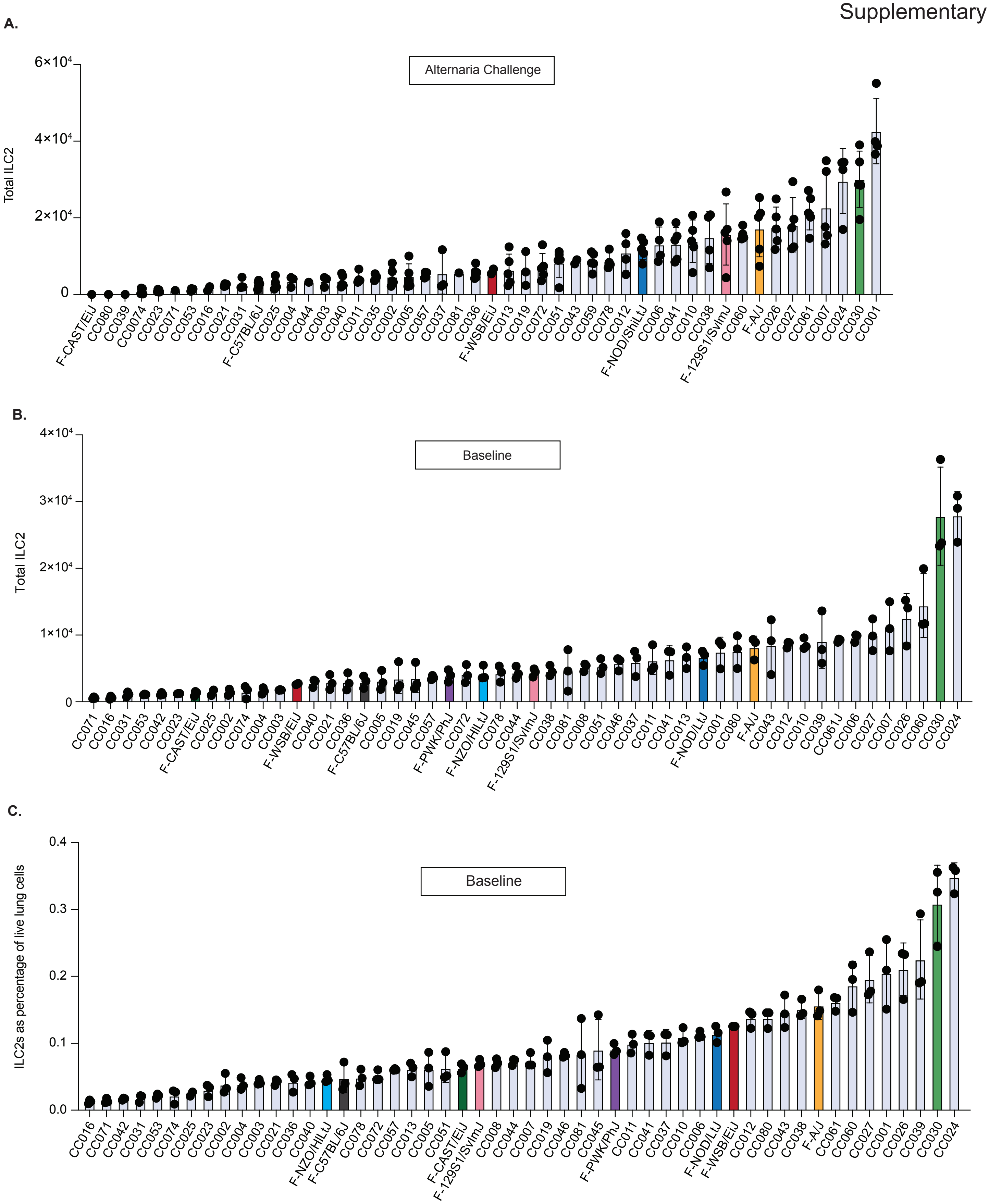

Supplementary Figure 2.

Strain differences amongst CC mice persist in total ILC2 numbers after challenge and in ILC2 prevalence and total numbers at baseline. (A) ILC2s from different CC strains quantified as total cells in the lung after *Alt* Ex challenge. Strains without bars indicate that all mice died during challenge. Colored bars represent founders and strains of interest. Bar height = mean, error bars = SD. Average n for strains challenged = 4.02. **(B)** ILC2s from different CC strains quantified as total cells in the lung at baseline without any challenge. Colored bars represent founders and strains of interest. Bar height = mean, error bars = SD. **(C)** ILC2s from different CC strains quantified as a percentage of live cells in the lung at baseline without any challenge. Colored bars represent founders and strains of interest. Bar height = mean, error bars = SD. *Alt* Ex challenged (A) and naïve (B) & (C) experiments were performed in separate batches of CC mice at different times. Average n for naïve strains = 2.96. Exact n for each strain provided in Source Data file.

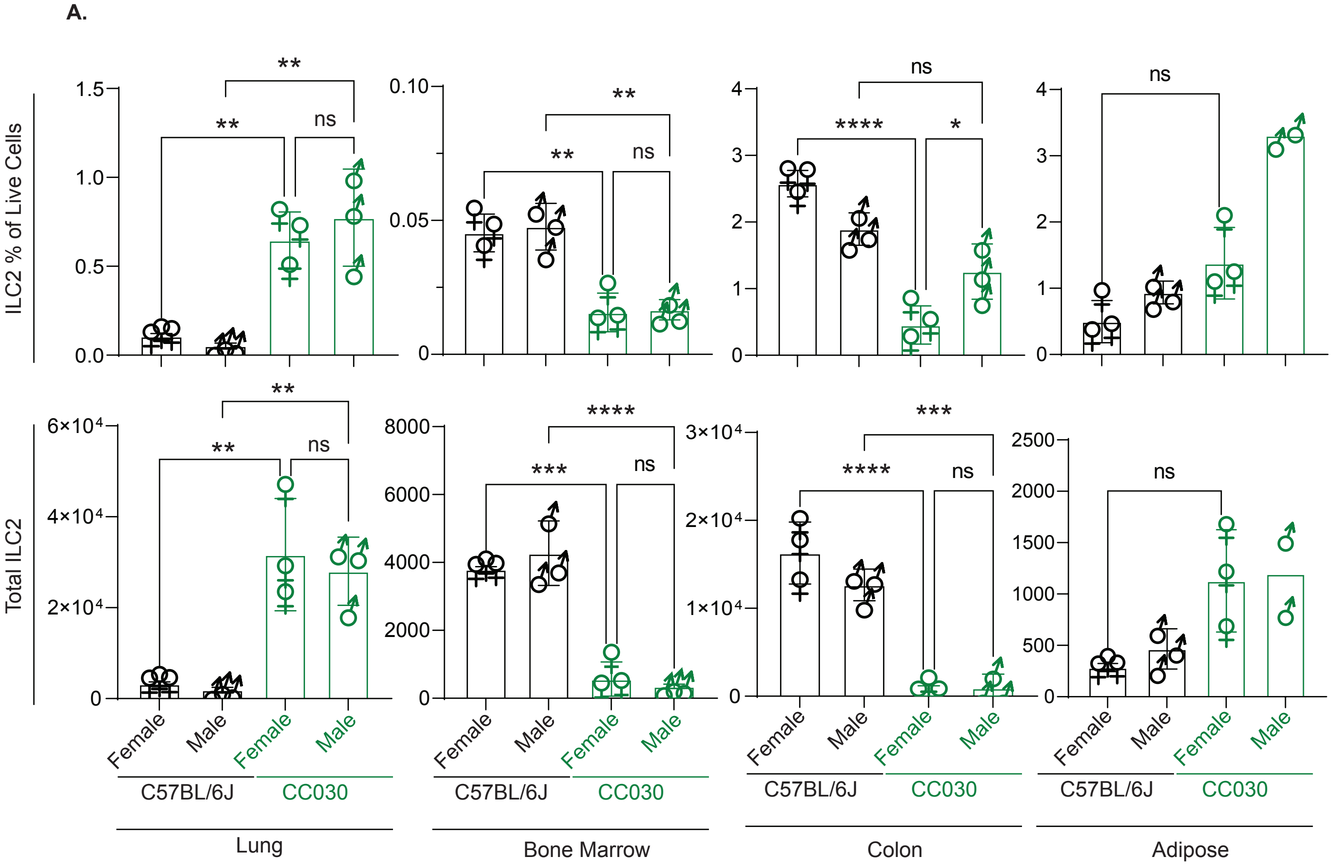
A.
ILC2 % of Live Cells
Total ILC2
**
ns
*
***
****
Female
Male
C57BL/6J
CC030
Lung
Bone Marrow
Colon
Adipose

Supplementary Figure 3.

The sex-independent increase of ILC2s in CC030 compared to C57BL/6J in the lung is not present in other tissues. (A) ILC2s are quantified in age matched, 8–10-week-old naïve males and females for both CC030 and C57BL/6J mouse strains. n=3 per strain and sex, adipose tissue was not recovered from 1 CC030 male. ILC2s for this tissue comparison experiment are defined as live singlet cells (FSC/SSC) that are Lineage-, CD90+, CD127+, and GATA3+. ILC2s as a percentage of live lung cells are calculated as a fraction of ILC2s over live singlet cells, multiplied by 100. Total ILC2 numbers are obtained with the addition of a known quantity of count beads to every sample. Result is representative of one independent experiment. Bar height = mean, error bars = SD. Significance was assessed by one-way ANOVA with Sidak's multiple comparisons testing. Exact p values are provided in the Source Data file.

Supplementary Figure 4

A.

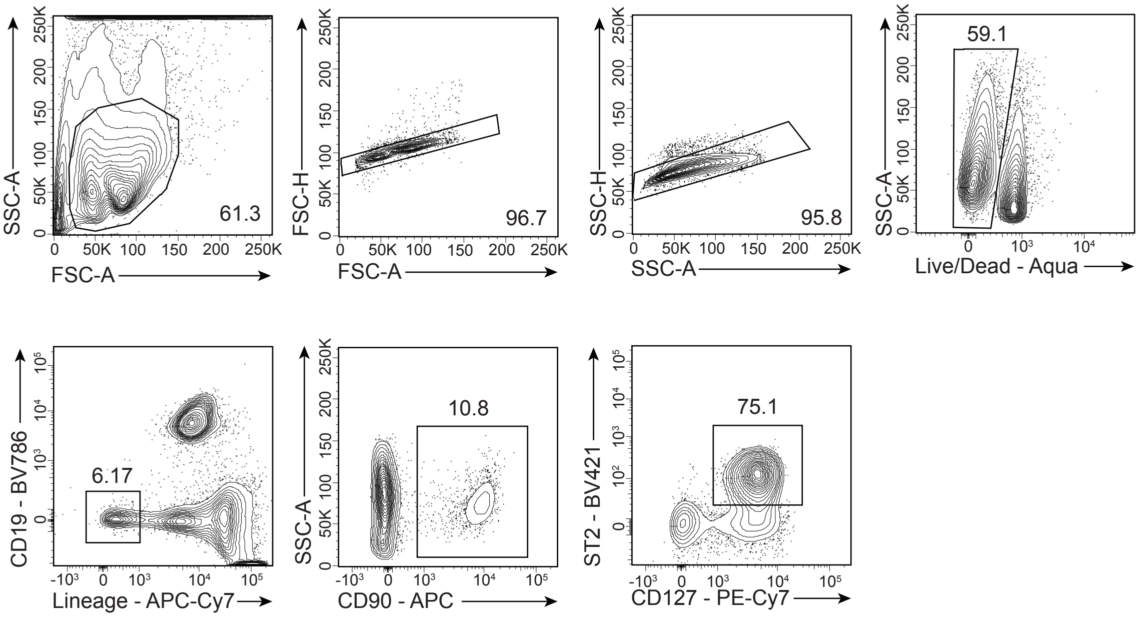

Supplementary Figure 4.

Flow cytometry sorting gating strategy for ILC2 culture. (A) Flow cytometry gating strategy for sorting lung ILC2s from naïve female CC030 mice for culture experiments with percentages.

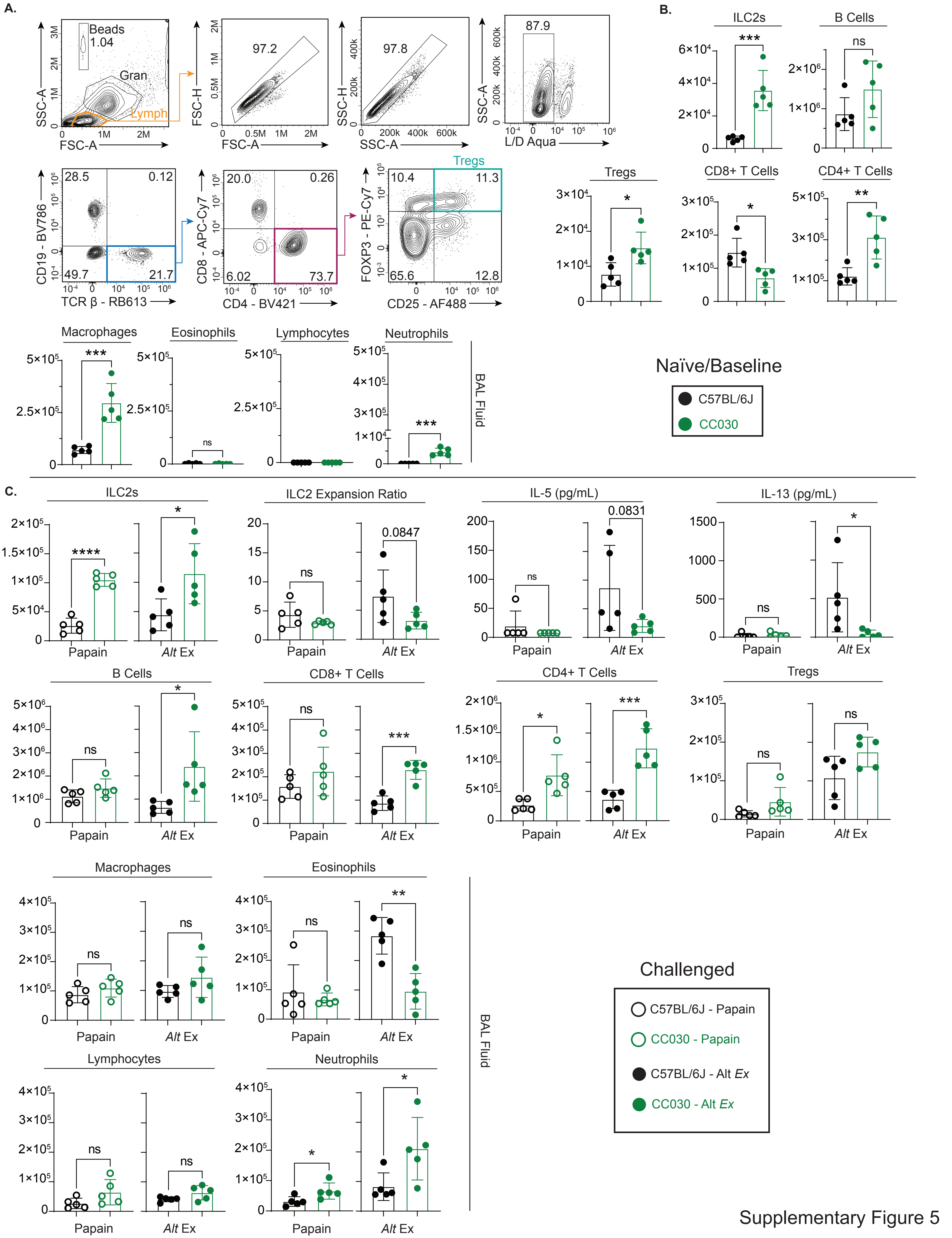

Supplementary Figure 5

Supplementary Figure 5.

CC030 mice have an increased neutrophilic and lymphocyte-driven response to aeroallergens compared to C57BL/6J. (A) Sample flow cytometry gating strategy for defining lymphocyte subsets. Lymph = lymphocytes, Gran = granulocytes, Treg = Regulatory T cells. **(B)** Various immune cell subsets present in C57BL/6J and CC030 mice at baseline isolated from the lung and analyzed by flow cytometry and enumerated in the BAL with hematoxylin and eosin (H&E) staining (n=5 for both strains, result is representative of one experiment). **(C)** Various immune cell subsets present in C57BL/6J and CC030 mice after airway challenge with *Alt* Ex (4-days, 8μg) and papain (3-days, 10μg). Bar height = mean, error bars = SD. Significance was assessed by two-tailed unpaired Student's t-test. Exact p values are provided in the Source Data file.

Supplementary Figure 6

A.

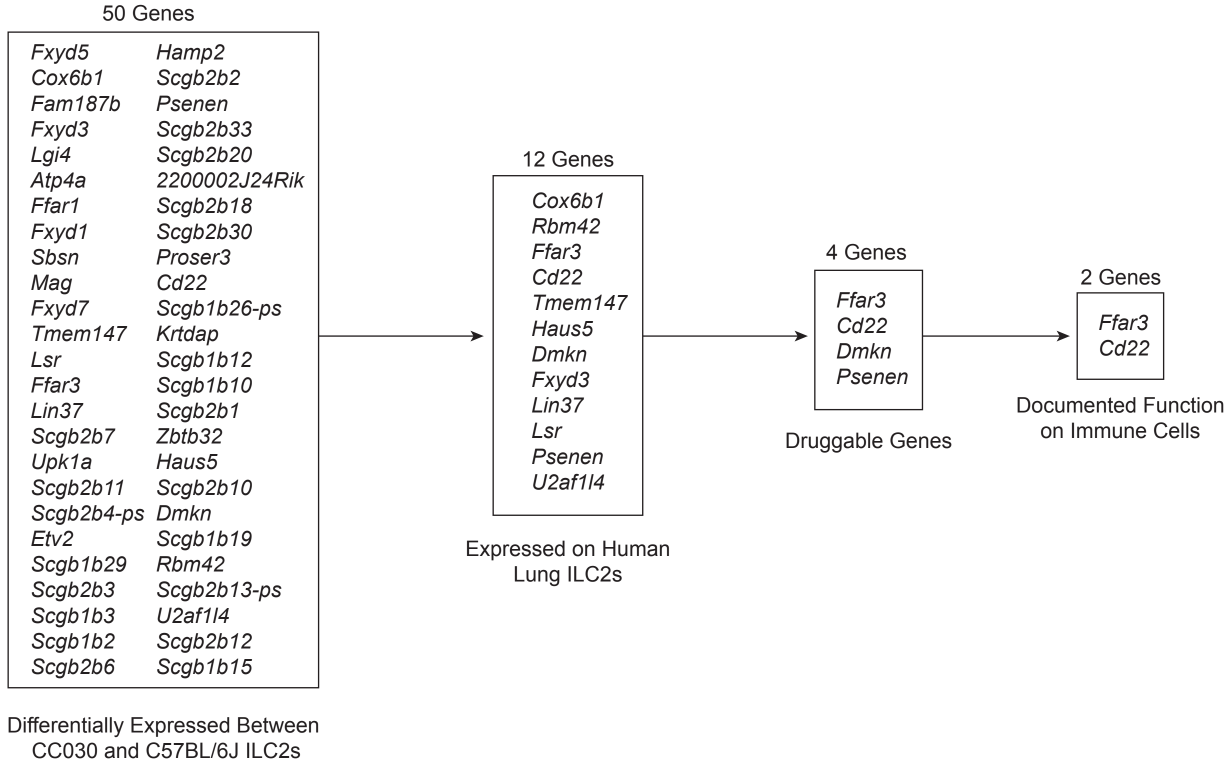

Supplementary Figure 6.

Detailed list of genes for each step in the selection of *Ffar3 and Cd22* as gene candidates. (A) A list of all QTL genes that are present after each selection step outlined in Figure 5 is provided. Protein coding genes were reduced to those differentially expressed between C57BL/6J and CC030 from the bulk-RNA sequencing described in Figure 3. Differentially expressed genes were narrowed to only those expressed on human ILC2s. Expressed genes were defined as those with a sum of normalized counts across 6 samples exceeding 10. Genes expressed on human ILC2s were narrowed to those genes represented in the druggable genome. Of 4 remaining genes, *Dmkn* and *Psenen* were excluded due to lack of previously documented evidence of function in immune cells. For the 12 genes labelled as expressed on human ILC2s, the names of mouse orthologs are provided. Assessment of whether *Ffar3*, *Cd22*, *Dmkn*, or *Psenen* had documented function on immune cells was assessed through PubMed searches for original research articles with the gene name and search term “immune”. Articles with experimental evidence for function of a gene on immune cells were considered positive results for selection.

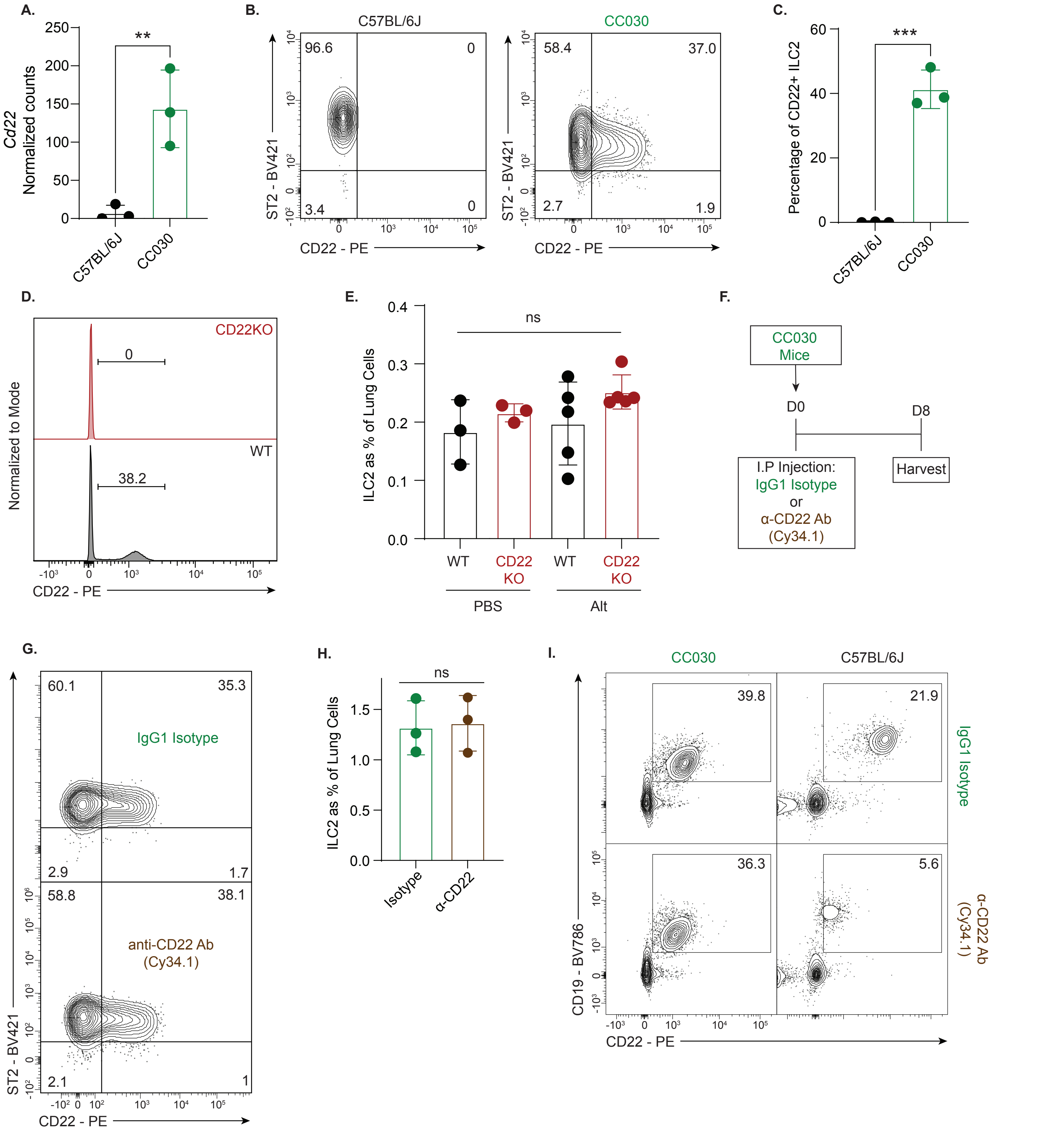
A.
Cd22
Normalized counts
250
200
150
100
50
0
**
C57BL/6J
CC030
B.
C57BL/6J
96.6
0
3.4
0
ST2 - BV421
CD22 - PE
CC030
58.4
37.0
2.7
1.9
ST2 - BV421
CD22 - PE
C.
60
40
20
0
***
Percentage of CD22+ ILC2
C57BL/6J
CC030
D.
CD22KO
0
WT
38.2
Normalized to Mode
CD22 - PE
E.
0.4
0.3
0.2
0.1
0.0
ns
ILC2 as % of Lung Cells
WT
CD22 KO
WT
CD22 KO
PBS
Alt
F.
CC030 Mice
D0
D8
I.P Injection:
IgG1 Isotype
or
α-CD22 Ab
(Cy34.1)
Harvest
G.
60.1
35.3
IgG1 Isotype
2.9
1.7
58.8
38.1
anti-CD22 Ab
(Cy34.1)
2.1
1
ST2 - BV421
CD22 - PE
H.
ns
1.5
1.0
0.5
0.0
ILC2 as % of Lung Cells
Isotype
α-CD22
I.
CC030
C57BL/6J
39.8
21.9
IgG1 Isotype
36.3
5.6
α-CD22 Ab
(Cy34.1)
CD19 - BV786
CD22 - PE

Supplementary Figure 7.

CD22 is expressed on CC030 ILC2s, but it is unlikely to regulate ILC2 numbers or effector function. (A) Normalized counts of *Cd22* for C57BL/6J and CC030 ILC2s from the bulk-RNA sequencing in Figure 3. Significance was obtained from the differential gene expression analysis used in DESeq2 with a Wald test. The p value has been corrected with the Benjamini-Hochberg method (** = $p<0.005$, n=3 per strain, result from one sequencing experiment). **(B)** Representative flow cytometry plots of CD22 expression on ILC2s from naïve female C57BL/6J and CC030 mice. Flow plots are pre-gated on ILC2s (live singlet cells (FSC/SSC) that are Lineage-, CD90+, CD127+, and GATA3+). **(C)** Percentage of lung ILC2s from CC030 and C57BL/6J that are positive for CD22, per the gating in (B) (n=3 per strain, result is representative of two independent experiments). Significance assessed with two-tailed unpaired Student's t-test. *** $p=0.0002$. **(D)** Representative flow plots assessing the specificity of α-CD22-PE (OX-97) used in (B). CD22 expression on live singlet cells in the lung is presented between WT C57BL/6J mice and CD22KO mice on the C57BL/6J background. **(E)** ILC2s as a percentage of live lung cells in WT and CD22KO mice (C57BL/6J background) in the 4-day *Alt* Ex challenge model (8μg) with PBS vehicle (n=3 for PBS groups, n=5 for *Alt* Ex groups, result representative of one experiment). Significance was assessed by one-way ANOVA. **(F)** Experimental protocol for treatment of CC030 mice with the α-CD22 blocking/depleting antibody Cy34.1 or IgG1 isotype control. **(G)** Representative flow cytometry plots of CD22 expression on ILC2s from unchallenged CC030 mice treated with α-CD22 (Cy34.1) blocking antibody or isotype control. **(H)** ILC2s as a percentage of live lung cells in CC030 mice treated with α-CD22 (Cy34.1) or isotype control (n=3 per group, result is representative of one experiment) Significance was assessed by two-tailed unpaired Student's t-test. **(I)** Representative flow cytometry plots of CD22 expression on CD19+ B cells in the lung of CC030 and C57BL/6J mice with frequency. Cells are pre-gated on live singlet cells. Bar height = mean, error bars = SD.

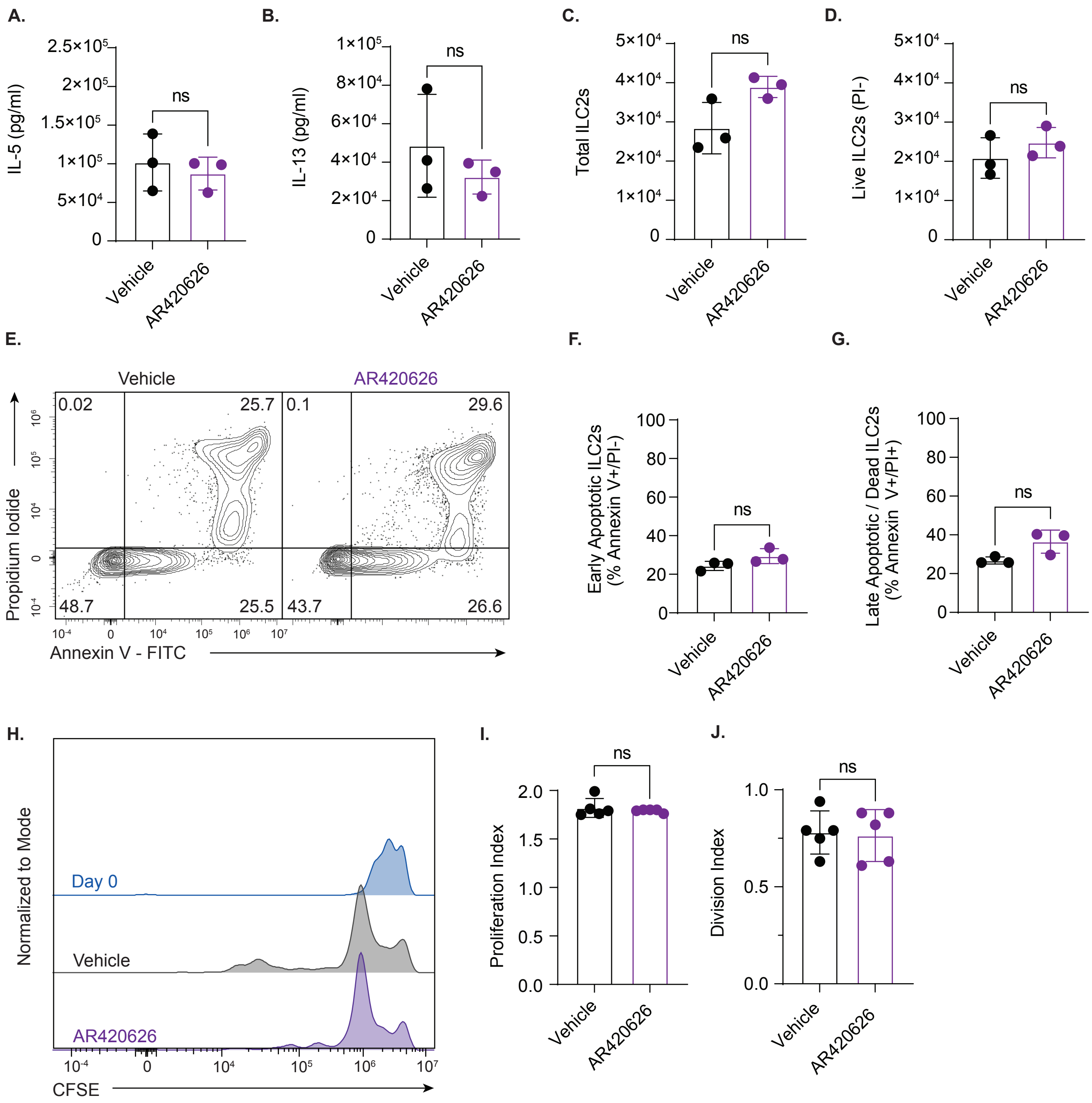
A.
IL-5 (pg/ml)
ns
Vehicle
AR420626
B.
IL-13 (pg/ml)
ns
C.
Total ILC2s
ns
D.
Live ILC2s (PI-)
ns
E.
Vehicle
AR420626
0.02
25.7
48.7
25.5
0.1
29.6
43.7
26.6
Propidium Iodide
Annexin V - FITC
F.
Early Apoptotic ILC2s (% Annexin V+/PI-)
ns
G.
Late Apoptotic / Dead ILC2s (% Annexin V+/PI+)
ns
H.
Normalized to Mode
Day 0
Vehicle
AR420626
CFSE
I.
Proliferation Index
ns
J.
Division Index
ns

Supplementary Figure 8.

FFAR3 agonism has no effect on Type 2 cytokine production, apoptosis, or proliferation in C57BL/6J ILC2s. A-G and H-J were performed as independent experiments on different batches of ILC2s isolated from C57BL/6J naïve female mice. All experiments use paired samples from distinct biological replicates. The experiments were performed with ILC2s cultured with IL-33 (10ng/mL), TSLP (10ng/mL), and IL-2 (100U/mL) +/- AR420626 (10µM) for 6 days (A-G) or 48 hours (H-J). **(A)** IL-5 in the supernatant (n=3). **(B)** IL-13 in the supernatant (n=3). **(C)** Total ILC2s (n=3). **(D)** Total live ILC2s (defined as PI-) (n=3). **(E)** Representative flow cytometry plot for assessment of early and late apoptosis by Annexin V and propidium iodide (PI) staining. Annexin V-/PI- = healthy cells, Annexin V+/PI- = early apoptotic cells, Annexin V+/PI+ = late apoptotic/dead cells. **(F)** Percentage of cells that are early apoptotic (Annexin V+/PI-) (n=3). **(G)** Percentage of cells that are late apoptotic/dead (Annexin V+/PI+) (n=3). **(H)** Representative flow plot of CFSE dilution experiment to measure proliferation after 48 hours of culture. **(I)** Proliferation and **(J)** Division Index to quantify proliferation between groups (n=5). Significance for each comparison was determined with a two-tailed paired Student's t-test. ns = not significant. Bar height = mean, error bars = SD.

A.

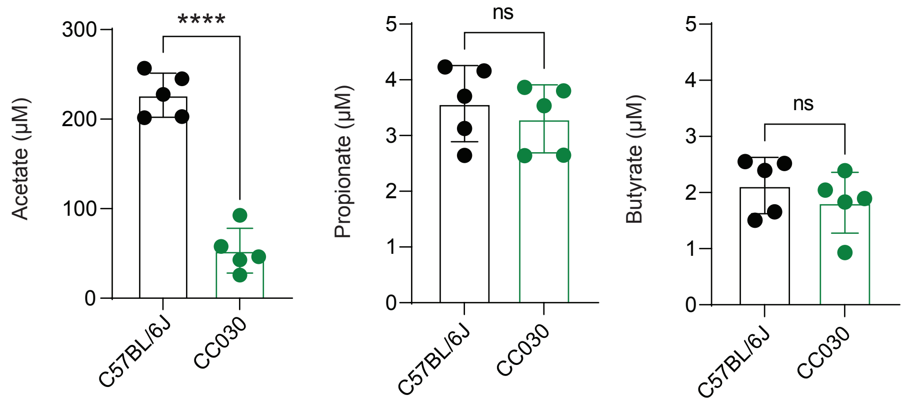
****
300
200
100
0
Acetate (µM)
C57BL/6J
CC030
ns
5
4
3
2
1
0
Propionate (µM)
C57BL/6J
CC030
ns
5
4
3
2
1
0
Butyrate (µM)
C57BL/6J
CC030

Supplementary Figure 9.

SCFA concentrations in the serum are different between C57BL/6J and CC030 mice at baseline. (A) Quantification of acetate, propionate, and butyrate in the serum of naïve C57BL/6J and CC030 female mice by mass spectrometry. Statistical significance was assessed with a two-tailed unpaired Student's t-test. n=5 per strain, result representative of 1 experiment. Bar height = mean, error bars = SD. ns = not significant, ****=p<0.0001.

Strain	Expansion Ratio
CC001	5.69
CC002	3.28
CC038	3.23
CC016	2.45
CC031	2.34
F-WSB/EiJ	2.26
CC004	2.14
CC025	2.11
F-A/J	2.1
CC041	2.07
CC078	2.06
CC007	2.02
CC003	1.93
CC036	1.92
CC019	1.87
CC071	1.84
CC027	1.82
CC072	1.74
CC010	1.61
CC051	1.56
CC005	1.5
CC057	1.41
CC026	1.4
CC006	1.35
CC012	1.26
CC040	1.23
CC081	1.21
CC053	1.11
CC060	1.09
CC030	1.08
CC024	1.06
CC043	1.0
CC013	0.96
CC037	0.92
F-C57BL/6J	0.88
CC021	0.85
CC023	0.77
CC044	0.74
CC011	0.7
CC080	0.0
F-CAST/EiJ	0.0
CC039	0.0

Supplementary Table 1.

Expansion ratios of ILC2s in the lungs of Collaborative Cross mice in response to *Alt* Ex challenge. Expansion ratios are calculated as the mean average of total ILC2s after *Alt* Ex challenge (provided in Supplementary Figure 2.A) divided by the mean average of total ILC2s at baseline (provided in Supplementary Figure 2.B) for each strain. Challenged and baseline experiments were done at different times with different batches of mice. Not all strains present in Figure 1.B and Supplementary Figure 2.A (Alternaria-challenged batch) had a second available batch of mice to use for naïve measurements, so no expansion ratio could be calculated for these strains.
