# Supplementary material for: Genetic diversity of Collaborative Cross mice implicates FFAR3 as a target for ILC2 anti-inflammatory reprogramming
Source: Nat Commun. 2026 Jan 3;17:1053. doi: 10.1038/s41467-025-67813-2 (PMC12847941; doi:10.1038/s41467-025-67813-2)
Supplement: Supplementary file 2 — Description of Additional Supplementary Files [file 41467_2025_67813_MOESM2_ESM.pdf]

Description of Additional Supplementary Files

Supplementary Data 1: Spreadsheet of differentially expressed genes between CC030 and C57BL/6J ILC2s

Supplementary Data 2: Spreadsheet of WikiPathways gene sets enriched in C57BL/6J ILC2s compared to CC030

Supplementary Data 3: Spreadsheet of Gene Ontology: Biological Processes gene sets enriched in C57BL/6J ILC2s compared to CC030

Supplementary Data 4: Spreadsheet of Gene Ontology: Biological Processes gene sets enriched in CC030 ILC2s compared to C57BL/6J

Supplementary Data 5: Spreadsheet of differentially expressed genes between AR420626 and DMSO vehicle treated ILC2s from CC030

Supplementary Data 6: Report of CRISPR-based *Ffar3* gene knockout attempt on CC030 mice

Supplementary Data 7: List of antibodies used in the presented experiments
