# Supplementary material for: Genetic diversity of Collaborative Cross mice implicates FFAR3 as a target for ILC2 anti-inflammatory reprogramming
Source: Nat Commun. 2026 Jan 3;17:1053. doi: 10.1038/s41467-025-67813-2 (PMC12847941; doi:10.1038/s41467-025-67813-2)
Supplement: Supplementary file 8 — Supplementary Data 6 [file 41467_2025_67813_MOESM8_ESM.pdf]

VGER Vanderbilt Genome Editing Resource

**Investigator**	Ray Stokes Peebles, M.D.<br>Professor of Medicine, Division of Allergy, Pulmonary and Critical Care Medicine
**Project start date**	September 2023
**Project termination date**	February 2024 (no live pups produced)
**Report prepared by**	Leesa Sampson, VGER Associate Director
**Report reviewed and approved by**	Kasia Jopek, VGER Research Specialist

Summary of results:

A collaborative cross recombinant inbred strain, derived from CC030/UncJ mice, with a targeted deletion of the *Ffar3* gene was attempted. Free fatty acid receptor 3 (Ffar3) is specifically expressed in innate lymphoid cells within this strain but not in other common mouse strains. The primary objective of this project is to elucidate the roles of Ffar3 in innate lymphoid cells.

Unfortunately, three CC030/UncJ pups with *Ffar3* deletions were found dead. The cause of their death remains unclear. Due to time constraints, the lead researcher—a Vanderbilt MSTP student—decided not to attempt to produce the desired allele again before departing the laboratory to complete medical school.

Genome editing strategy:

Ffar3 is located within a flanking genomic interval on chromosome 7 (coordinates 30,835,532 to 30,861,261) that is entirely contributed by the CAST/EiJ strain. This information was obtained using online tools available at the following link: https://csbio.unc.edu/CCstatus/index.py?run=locus.

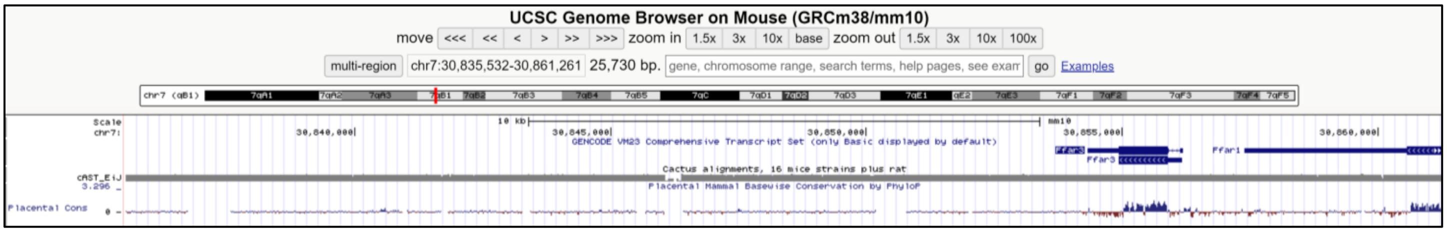

Guide RNAs were selected to flank the majority of the *Ffar3* coding sequence, as depicted below. By deleting the DNA between these two sites, with precision aided by the inclusion of a short single-stranded DNA containing an XhoI restriction site and homology arms matching the flanking DNA, most of the *Ffar3* coding sequence is lost. This *Ffar3* deletion can be identified through PCR-based analysis by the presence of a 331 bp PCR product.

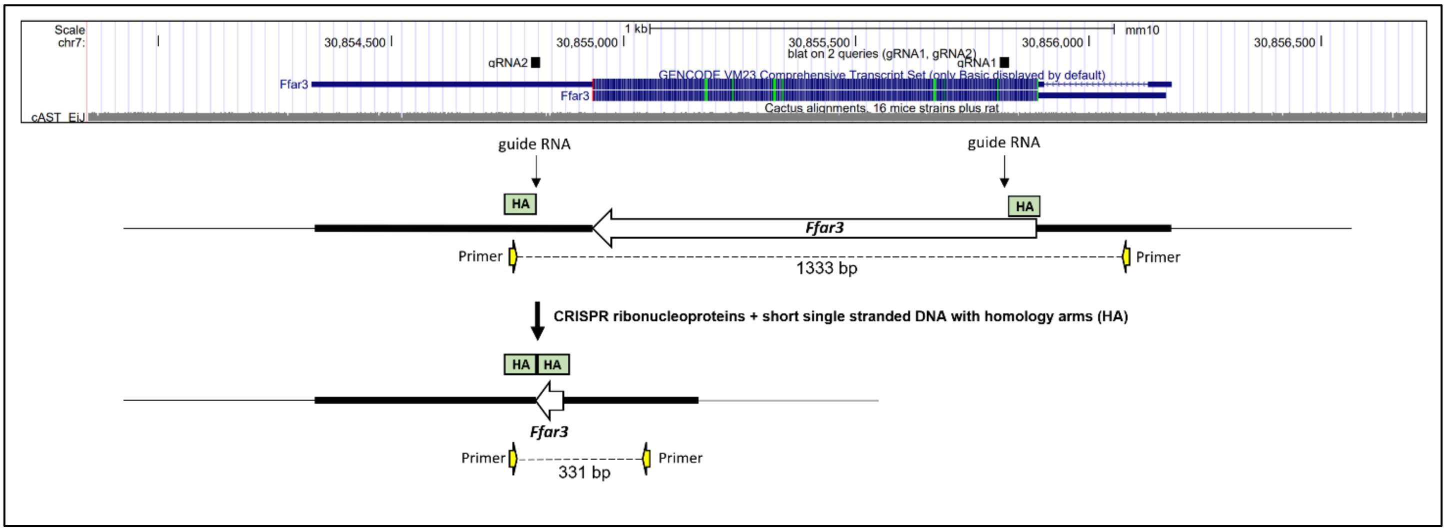

Production of genome edited mice:

The CC030/UncJ mouse zygotes were electroporated with assembled ribonucleoproteins containing HiFi Cas9 protein and guide RNAs mixed with a single stranded DNA donor oligonucleotide (for a templated *Ffar3* deletion) and subsequently transferred to the oviducts of CD-1 surrogate dams.

Key resources table:

Reagent	Source	Product Identifier
Alt-R® CRISPR-Cas9 crRNA	IDT	Ffar3KO_CDS<br>CGTTGAGGGGGAGTCCCACG
Alt-R® CRISPR-Cas9 crRNA	IDT	Ffar3KO_3'<br>ACTCTAGTCCGTCTGAGCTA
Alt-R® CRISPR-Cas9 tracrRNA	IDT	#1072532
Alt-R™ S.p. HiFi Cas9 Nuclease V3, 100 µg	IDT	#1081060
Alt-R® HDR Donor Oligo	IDT	Ffar3KOssDNA<br>CCATGGGGACAAGCTTCTTTCTTGGCAATTACTGGCTTTTC<br>TTTTCCGTGTACCTGTTGGTGTTCCTCGTCTCGAGCTCAG<br>ACGGACTAGAGTGCACTGTGACAGGCTGCAGACATTGCT<br>GTCACTCAGATTGCCCTGAGTGCACT

Results of attempt to delete *Ffar3* in CC030/UncJ mice:

Total embryos recovered	Total embryos edited and transferred	Pseudopregnant female transfers	Dead pups screened	Live pups produced
244	108 (44%)	5	3 (2.8%)	0

In our facility, we typically perform four embryo transfers per project. On average, approximately 20% of the transferred embryos result in live pups, assuming that genome modifications do not adversely affect development. Visible pregnancy was observed in four out of the five dams. Unfortunately, only three deceased pups were recovered upon reaching their due date, which is significantly lower than the expected average.

The advanced state of decomposition observed in the deceased pups suggests death may have occurred prior to delivery. To investigate further, the two dams housed in the same cage as the deceased pups were sacrificed and examined for implantation sites. One female had four implantation sites, while the other had two. This discrepancy suggests the loss of additional embryos after implantation, although no corresponding tissue was recovered. The remaining three surrogates that failed to deliver any pups were not subjected to further examination.

Tissues from the three dead pups were collected and screened by PCR for evidence of *Ffar3* deletions:

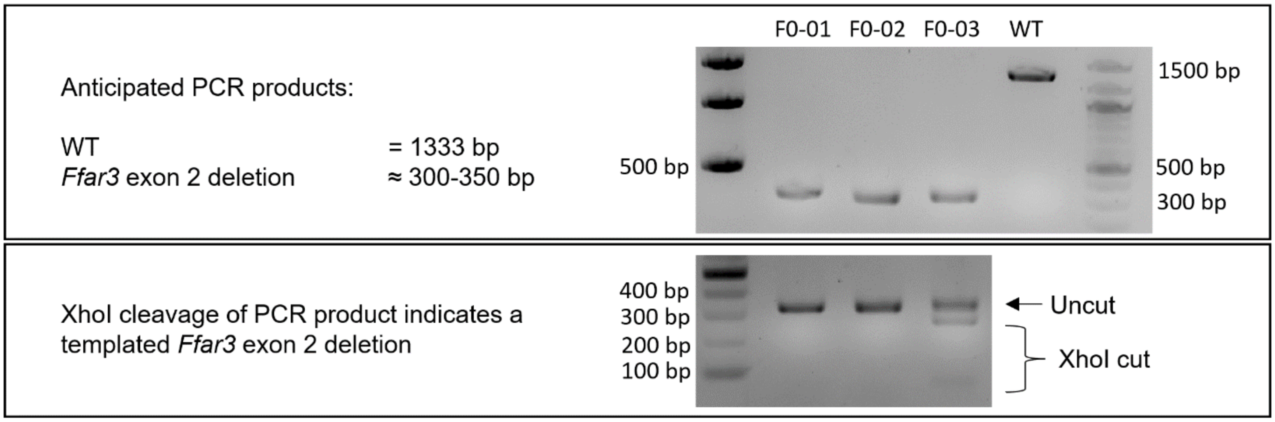

All three pups tested positive for *Ffar3* deletion alleles. Pup IDs F0-01 and F0-02 exhibited untemplated deletions between the two guide RNA sites, a common occurrence in CRISPR editing. In contrast, F0-03 displayed a precise, templated deletion as originally designed, indicating successful execution of the *Ffar3* deletion genome editing strategy. However, the exact percentage of *Ffar3* knockout tissue contribution in the F0 mice remains unknown due to their mosaic nature. It is likely that they also harbor other undetected *Ffar3* mutant or wild-type alleles.

Without further analysis, the cause of pup mortality remains uncertain. Potential factors include genetic effects from on-target *Ffar3* deletion, off-target edits, or environmental influences.

1. According to IMPC data, homozygous *Ffar3* knockout mouse in the C57BL/6NCrl strain are produced in expected ratios:

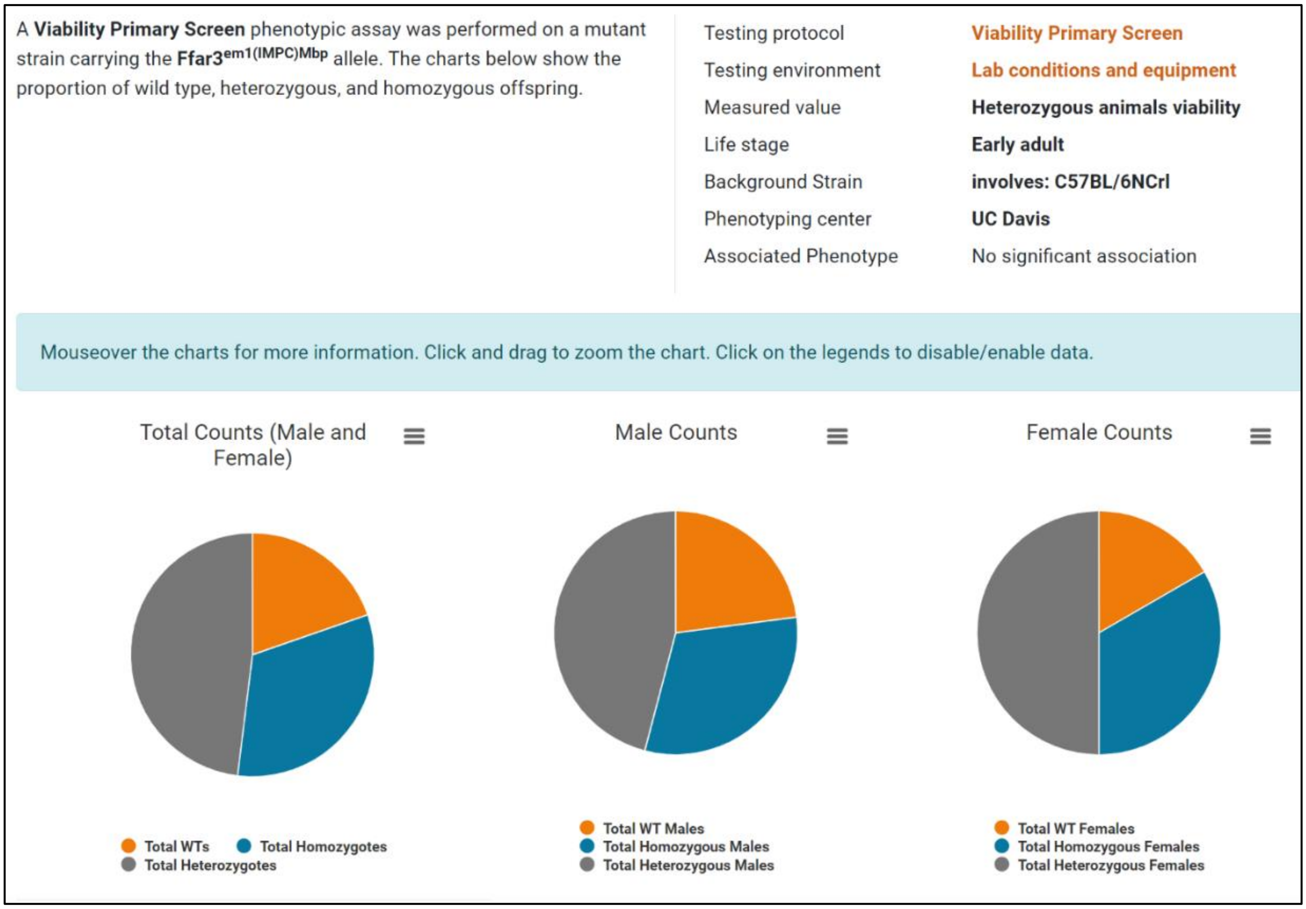

2. Off-target edits are a potential concern when using CRISPR/Cas genome editing. In our laboratory, we employ a high-fidelity (HiFi) SpCas9 protein from IDT, which is known to reduce the likelihood of off-target edits: [A high-fidelity Cas9 mutant delivered as a ribonucleoprotein complex enables efficient gene editing in human hematopoietic stem and progenitor cells (researchgate.net)](). Additionally, we carefully select guide RNAs with low off-target probabilities based on computational models that reference the C57BL/6J strain genome.

   However, our standard approach for predicting off-target edits is not applicable to the CC030/UncJ strain. This strain is composed of genetic contributions from eight inbred strains, making it more complex than the typical C57BL/6J background. Consequently, we cannot definitively rule out the possibility of off-target editing affecting a critical gene, potentially contributing to the observed lethality in the *Ffar3*-edited pups.

3. Environmental factors can significantly influence birth rates. Our nursery is located within a private room in the MCN barrier facility. Changes in staffing, humidity levels, ambient sound, and odors can all impact pregnancy and pup survival after birth. The Department of Animal Care takes every possible measure to control these external factors, but occasionally, we still encounter periods of lower-than-average pup numbers.

Summary statement:

These data suggest that the *Ffar3* knockout strategy was highly efficient, as all three deceased pups carried potentially suitable edited alleles. Regrettably, live pups were not recovered, and time constraints prevented the lead investigator from again attempting to produce this strain. The cause of the atypical pup mortality rate remains unknown.
