# Supplementary material for: Genetic diversity of Collaborative Cross mice implicates FFAR3 as a target for ILC2 anti-inflammatory reprogramming
Source: Nat Commun. 2026 Jan 3;17:1053. doi: 10.1038/s41467-025-67813-2 (PMC12847941; doi:10.1038/s41467-025-67813-2)
Supplement: Supplementary file 10 — Reporting Summary [file 41467_2025_67813_MOESM10_ESM.pdf]

nature portfolio

Corresponding author(s): Mark Rusznak, R. Stokes Peebles, Jr.

Last updated by author(s): Nov 24, 2025

Reporting Summary

Nature Portfolio wishes to improve the reproducibility of the work that we publish. This form provides structure for consistency and transparency in reporting. For further information on Nature Portfolio policies, see our Editorial Policies and the Editorial Policy Checklist.

Statistics

For all statistical analyses, confirm that the following items are present in the figure legend, table legend, main text, or Methods section.

n/a	Confirmed	
☐	☒	The exact sample size (*n*) for each experimental group/condition, given as a discrete number and unit of measurement
☐	☒	A statement on whether measurements were taken from distinct samples or whether the same sample was measured repeatedly
☐	☒	The statistical test(s) used AND whether they are one- or two-sided<br>*Only common tests should be described solely by name; describe more complex techniques in the Methods section.*
☐	☒	A description of all covariates tested
☐	☒	A description of any assumptions or corrections, such as tests of normality and adjustment for multiple comparisons
☐	☒	A full description of the statistical parameters including central tendency (e.g. means) or other basic estimates (e.g. regression coefficient) AND variation (e.g. standard deviation) or associated estimates of uncertainty (e.g. confidence intervals)
☐	☒	For null hypothesis testing, the test statistic (e.g. *F*, *t*, *r*) with confidence intervals, effect sizes, degrees of freedom and *P* value noted<br>*Give P values as exact values whenever suitable.*
☒	☐	For Bayesian analysis, information on the choice of priors and Markov chain Monte Carlo settings
☒	☐	For hierarchical and complex designs, identification of the appropriate level for tests and full reporting of outcomes
☒	☐	Estimates of effect sizes (e.g. Cohen's *d*, Pearson's *r*), indicating how they were calculated

Our web collection on statistics for biologists contains articles on many of the points above.

Software and code

Policy information about availability of computer code

Data collection	No special software was used for data collection.
Data analysis	All statistical analysis outside of QTL mapping and transcriptomic analysis was carried out in GraphPad Prism 10 software (version 10.6.1). QTL mapping was performed at each SNP using the qtl2 package in R (version 4.2.1). Sequencing data quality was initially assessed using FastQC (version 0.12.1), followed by read trimming with fastp (version 1.0.1). For the C57BL/6J samples, reads were aligned to the mm10 reference genome using STAR (version 2.7.11b) with default parameters. For the CC030 samples, reads were aligned to the mm9 reference pseudogenome using STAR with default parameters, after which lapels (version 1.1.1) was used to convert the alignments to mm10 reference coordinates. Gene expression counts were subsequently quantified across all samples using HTSeq (version 2.0.5). Differential gene expression analysis was performed on the resultant read count matrices using the DESeq2 (version 3.22) R package. Normalized counts of genes, sample distance matrices, and principal component analyses were conducted with DESeq2, and results were visualized with ggplot2 (version 3.5.2) and pheatmap (1.0.13). Differentially expressed genes were visualized in volcano plots with EnhancedVolcano (version 1.22.0). Mouse genes were converted to human orthologs with biomaRt (version 2.60.1). ClusterProfiler (version 4.12.6) was used to perform gene-set enrichment analysis using Gene Ontology: Biological Processes and WikiPathways curated gene sets and to generate the resultant tree plot. For the PCA plot in Figure 6, values in the normalized count matrices were log2-transformed to stabilize variance. We corrected for inter-experiment batch effects with ComBat from the sva R package (version 3.52.0).

For manuscripts utilizing custom algorithms or software that are central to the research but not yet described in published literature, software must be made available to editors and reviewers. We strongly encourage code deposition in a community repository (e.g. GitHub). See the Nature Portfolio guidelines for submitting code & software for further information.

Data

Policy information about availability of data

All manuscripts must include a data availability statement. This statement should provide the following information, where applicable:

- Accession codes, unique identifiers, or web links for publicly available datasets
- A description of any restrictions on data availability
- For clinical datasets or third party data, please ensure that the statement adheres to our policy

Original high-throughput RNA-sequencing data was generated for this manuscript as described in the methods. The metadata file, processed read-count matrix data, and raw data are available on GEO, with the accession number: GSE288176. The reviewer token is: mnafwkeirtmdnmr. Source Data are provided with this paper. All other data represented in this manuscript are available to any interested party upon reasonable request.

Research involving human participants, their data, or biological material

Policy information about studies with human participants or human data. See also policy information about sex, gender (identity/presentation), and sexual orientation and race, ethnicity and racism.

Reporting on sex and gender	Participants of any sex were eligible and enrolled under the IRB-approved PBMC donation protocol, and we were informed that both male and female donors were included. However, individual-level sex information was not provided to the investigators performing cell isolation, culture, or downstream analyses, as samples were fully de-identified prior to transfer. As a result, sex-disaggregated analyses could not be performed.
Reporting on race, ethnicity, or other socially relevant groupings	Race and ethnicity information was collected under the IRB-approved protocol and was self-reported by participants. However, individual-level race/ethnicity data were not provided to the investigators performing cell isolation, culture, or downstream analyses, as all samples were fully de-identified prior to transfer.
Population characteristics	The average age for participants in this study was 34.5 years. All participants in this study had the following characteristics: non-pregnant individuals over the age of 18 who do not have asthma. Exclusion criteria for all participants: participants with a viral or bacterial infection at the time of the study, participants weighing less than 110lbs, women who were pregnant, breastfeeding, were on estrogen replacement therapy, were taking hormonal birth control medications, had had a hysterectomy, or were menopausal.
Recruitment	Investigators used email advertisement through Vanderbilt University Medical Center and ResearchMatch.com, an online database connecting researchers to study participants, for recruitment of healthy individuals and participants with asthma. To recruit patients with asthma from the Vanderbilt Asthma, Sinus, and Allergy (VASAP) Clinic at Vanderbilt University Medical Center, research personnel requested that physicians identify patients in their clinics that would qualify for the study. Research personnel will also look at the upcoming severe asthma clinic schedule at VASAP to identify patients that may also qualify for the study. Whenever an eligible patient is identified, the patient will be contacted by research personnel to discuss the possibility of the patient's inclusion in the study. While the study recruited patients with varying asthma status, only individuals who did not have asthma were used for this manuscript.
Ethics oversight	Vanderbilt University Medical Center Institutional Review Board - IRB# 202162

Note that full information on the approval of the study protocol must also be provided in the manuscript.

Field-specific reporting

Please select the one below that is the best fit for your research. If you are not sure, read the appropriate sections before making your selection.

[x] Life sciences [ ] Behavioural & social sciences [ ] Ecological, evolutionary & environmental sciences

For a reference copy of the document with all sections, see nature.com/documents/nr-reporting-summary-flat.pdf

Life sciences study design

All studies must disclose on these points even when the disclosure is negative.

Sample size	Sample sizes for individual Collaborative Cross recombinant strains tested in Figure 1 was determined from previously published estimates (Keele et al., 2019, PMID: 30914424). Sample sizes for other experiments were based off of previous studies from our lab which used similar experimental endpoints.
Data exclusions	One data exclusion exists in Supplementary Figure 3, panels B and C. In this in vitro ILC2 culture experiment, two wells that were located on the edge of the 96-well U-bottom non-tissue culture treated plate experience significant evaporation (less than 50% of volume of other wells). Due to this increased evaporation, those two replicates were excluded from analysis. 1 excluded replicate came from the "Vehicle" group, and the other came from the "10μM" group.
Replication	RNA sequencing experiments and phenotyping of the 48 Collaborative Cross recombinant and founder strains were performed once. Mouse in vitro and in vivo studies were performed at least 2 times. Experiments for Figure 5 B, C, F, H, and I were performed 4 times. Data from individual human participants was represented together from 3 separate experimental days. Replication of individual human samples was not

	performed due to the de-identified nature of samples being given to the investigators performing the culture experiments. All attempts at replication were successful.
Randomization	Animal subjects in mouse studies were not randomized due to experiments comparing strain or genotype differences. All mice were age matched and kept in identical housing conditions to control for variation. In vitro studies used either pooled cells from multiple biological replicates or different treatments on cells from the same biological replicate, allowing for paired comparisons.
Blinding	The investigator performing the cell differential on bronchoalveolar lavage (BAL) fluid was blinded to the groups. Given that all other data being gathered was quantitative and obtained through an instrument, blinding was not deemed necessary.

Reporting for specific materials, systems and methods

We require information from authors about some types of materials, experimental systems and methods used in many studies. Here, indicate whether each material, system or method listed is relevant to your study. If you are not sure if a list item applies to your research, read the appropriate section before selecting a response.

Materials & experimental systems

n/a	Involved in the study
☐	☒ Antibodies
☒	☐ Eukaryotic cell lines
☒	☐ Palaeontology and archaeology
☐	☒ Animals and other organisms
☒	☐ Clinical data
☒	☐ Dual use research of concern
☒	☐ Plants

Methods

n/a	Involved in the study
☒	☐ ChIP-seq
☐	☒ Flow cytometry
☒	☐ MRI-based neuroimaging

Antibodies

Antibodies used	Lineage Streptavidin 130-092-613 5230605119 Miltenyi, CD3 Streptavidin 17A2 100244 B349600 BioLegend, CD4 Streptavidin GK1.5 100404 B365003 BioLegend, FcεR1 Streptavidin MAR-1 134304 B274187 BioLegend, CD45 AF700 30-F11 56-0451-82 2211060 Invitrogen, ICOS BB515 C398.4A 565880 1348425 BD, CD25 BV786 PC61 564023 2038819 BD, CD127 PE-Cy7 SB/199 560733 1159448 BD, GATA3 APC TWAJ 50-9966-42 2430297 Invitrogen, CD90.2 APC 53-2.1 140312 B364723 BioLegend, ST2 BV421 U29-93 566309 2238614 BD, GATA3 PE-CF594 L50-823 563510 335611 BD, CD25 AF488 PC61 102017 B380907 BioLegend, CD22 PE OX-97 126112 B315505 BioLegend, CD19 BV786 1D3 563333 4003786 BD, CD22 Cy34.1 BE0011 779021A2 BioXCell, Isotype MOPC-21 BE0083 78512101 BioXCell, IL-5 APC TRFK5 554396 1265316 BD, IL-13 PerCP-eF710 eBio13A 46-7133-80 2727209 Invitrogen, Ki-67 PE SolA15 12-5698-80 4278633 Invitrogen, EGFR H11 MA5-13070 YK4137801 Invitrogen, Isotype Super Bright 436 eBR2a 62-4321-80 2082341 Invitrogen, Isotype BB515 A19-3 564460 9220598 BD, Isotype BV786 R35-95 563335 5128556 BD, Isotype PE-Cy7 A95-1 552849 7011928 BD, Isotype APC MPC-11 400319 B202284 BioLegend, Lineage (CD3/14/16/19/20/56) FITC 348801 B404928 BioLegend, CD45 Super Bright 780 2D1 78-9459-42 2764005 Invitrogen, CD127 PE-Cy7 A019D5 351320 B406035 BioLegend, CRTH2 APC/Cy7 BM16 350114 B388629 BioLegend, EGFR APC AY13 352906 B424773 BioLegend, Isotype APC MOPC-21 400120 B404487 BioLegend, TCR-B RB613 H57-597 758429 5027686 BD, CD4 BV421 GK15 100437 B297643 BioLegend, CD8a APC-Cy7 53-6.7 557654 4064668 BD, FFAR3 FITC FFAR3-FITC 2440.P1d.Ig.20 FabGenix, FFAR3 1D10B7 66811-1-Ig 10007183 proteintech, Goat anti-mouse IgG AF488 A28175 Invitrogen, IL-10 JES052A5 MAB417 AHZ0621051 R&D, Isotype 43414 MAB005 R&D, Goat IgG Isotype 02-6202 Invitrogen, Amphiregulin AF989-SP R&D FOXP3 PE-Cy7 FJK-16s 25-5773-82 2254250 invitrogen, IL-13 PE-Cy7 eBio13A 25-7133-82 2011693 Invitrogen.
Validation	Antibodies used to detect CD22 on ILC2s (CD22 PE OX-97 126112 B315505 BioLegend) were validated against knockout mice (CD22KO, Extended Data Figure 5.D) to ensure specificity. EGFR expression on mice was assessed with EGFR (H11 MA5-13070 YK4137801 Invitrogen) and validated with secondary only controls and isotype controls. All other antibodies had sufficient validation provided by the manufacturer.

Animals and other research organisms

Policy information about studies involving animals; ARRIVE guidelines recommended for reporting animal research, and Sex and Gender in Research

Laboratory animals	All Collaborative Cross (CC) recombinant strains used in this manuscript (CC001/Unc, 002/Unc, 003/Unc, 004/TauUnc, 005/TauUnc, 006/TauUnc, 007/Unc, 010/GeniUnc, 011/Unc, 012/GeniUnc, 013/GeniUnc, 016/GeniUnc, 019/TauUnc, 021/Unc, 023/GeniUnc, 024/GeniUnc, 025/GeniUnc, 027/GeniUnc, 030/GeniUnc, 031/GeniUnc, 035/Unc, 036/Unc, 037/TauUnc, 038/GeniUnc, 039/Unc, 040/TauUnc, 041/TauUnc, 043/GeniUnc, 044/Unc, 051/TauUnc, 053/Unc, 057/Unc, 059/TauUnc, 060/Unc, 061/GeniUnc, 071/TauUnc, 072/TauUnc, 074/Unc, 078/TauUnc, 080/TauUnc, 081/Unc) were obtained from the UNC Systems Genetics Core Facility. CC founder strains 129S1/SvlmJ, A/J, C57Bl/6J, CAST/EiJ, NOD/ShiLtJ, and WSB/EiJ mice were obtained from Jackson Laboratories. C57BL/6-Cd22tm1Lam /J (CD22KO) mice were obtained from Jackson Laboratories. CC030 mice, C57BL/6J, and CD22KO mice were bred and maintained in our mouse colony. All In vivo and In vitro mouse experiments utilized age-matched mice 8-16 weeks old.
Wild animals	No wild animals were used for this study.

Reporting on sex	Female mice were used in the primary experiments of this paper. The major findings of ILC2 prevalence in the Collaborative Cross strain of interest (CC030/GeniUnc) were validated in age-matched male mice to determine if the effect was sex-dependent or applicable to male and female. Mouse sex was determined by inspection of the gonads at 4 weeks of age.
Field-collected samples	No samples were collected from the field.
Ethics oversight	Ethical oversight for animal experiments was provided by the Vanderbilt University Medical Center Institutional Animal Care and Use Committee (IACUC). The IACUC protocol used for the experiment is M1800150-01 (PI: Ray Peebles).

Note that full information on the approval of the study protocol must also be provided in the manuscript.

Plants

Seed stocks	No seed stocks were used in this study.
Novel plant genotypes	No novel plant genotypes were produced during this study.
Authentication	No seed stocks were used in this study. Therefore, no authentication was performed.

Flow Cytometry

Plots

Confirm that:

- [x] The axis labels state the marker and fluorochrome used (e.g. CD4-FITC).
- [x] The axis scales are clearly visible. Include numbers along axes only for bottom left plot of group (a 'group' is an analysis of identical markers).
- [x] All plots are contour plots with outliers or pseudocolor plots.
- [x] A numerical value for number of cells or percentage (with statistics) is provided.

Methodology

Sample preparation	Mouse lungs were placed in a digestion solution (RPMI 1640, 5% FBS, 1mg/mL Type IV Collagenase from Clostridium histolyticum (Sigma), and 20 µg/mL of DNase I) and minced with scissors. Lungs were digested for 35 minutes at 37°C with rotation. Digestion was neutralized with EDTA, and lungs were subsequently ground through cell strainers to obtain a single cell suspension. Mouse bone marrow was obtained from the femurs and tibias of mice after euthanasia. Bones were cleared of muscle and connective tissue, and the ends of the long bones were cut with a scalpel. Bones were placed into 0.5mL tubes with puncture holes in the bottom. These punctured tubes were fit into 1.5mL tubes and centrifuged, allowing for the collection of the bone marrow effluent. Bone marrow cells were then passed through cell strainers. Gonadal adipose tissue was excised bilaterally from the mice after euthanasia. Adipose tissue was minced and suspended in digestion buffer (2mg/mL Collagenase (Sigma C-6885) with 1% FBS in PBS). Digestion was carried out at 37°C with rotation for 45 minutes. Digested adipose tissue was passed through a cell strainer and diluted with neutralization buffer (2mM EDTA with 1% FBS in PBS). Samples were centrifuged, and the supernatant with adipocytes was removed. Colons were harvested from mice and washed with cold PBS. Colons were then opened longitudinally and cut into 0.5cm pieces, which were subsequently incubated with RPMI containing Penicillin/Streptomycin (Pen/Strep), 5mM EDTA, 20mM HEPES, 5% FBS and 1mM DTT for 40 minutes at 37°C with shaking. Contents were then poured through cells strainers, with the flowthrough discarded. Remaining tissue pieces were shaken vigorously in cold RPMI with Pen/Strep, 5mM EDTA, and 20mM HEPES and passed through a cell strainer, again discarding flowthrough. Tissue pieces were minced in a beaker and incubated in RMPI Pen/Strep, 5mM EDTA, and 20mM HEPES, 0.1 mg/mL Liberase TL (Roche), 0.05% DNase I (Sigma D5025) with stirring for 30 minutes at 37°C. Contents were pulled through a 10mL syringe and poured through a cell strainer. Cells were subsequently washed with RPMI with Pen/Strep, 5mM EDTA, and 20mM HEPES, and 0.05% DNase. Cells were then isolated with a 40%/90% Percoll gradient. Erythrocyte lysis was carried out for all samples with ammonium chloride-based RBC lysis solution (Tonbo™) for 5 minutes before neutralization with cold PBS. Analytical flow cytometry from mouse tissues: Viability staining was carried out in PBS with Live/Dead Aqua Fixable Viability dye. Surface staining for single cell suspensions was carried out in FACS staining buffer (3% FBS in PBS). Prior to staining, cells were incubated with Fc block for 10 minutes. Surface staining for ILC2 was carried out with Lineage staining cocktail (α-CD3, α-CD4, α-FCεR1, Hematopoietic Lineage Labeling Cocktail, anti-mouse biotin (Miltenyi™)), α-CD19, α-CD45, α-CD90, α-CD127, α-ST2, α-ICOS, α-CD25, α-CD22 for 20 minutes. Streptavidin conjugation to biotinylated antibodies was carried out for 15 minutes. Fixation and permeabilization was carried out overnight with the FoxP3 Transcription Factor Staining Buffer Kit (eBioscience™). Intracellular staining for GATA3 was carried out for 1 hour in PermBuffer (eBioscience™). Analysis was conducted in FACS staining buffer on a BD 5-Laser Fortessa. Flow Cytometry Sorting: Single cell suspensions were obtained from the naïve lungs of mice according to the harvest procedure outlined in this reporting summary. For the flow sorting experiment described in Figure 4, ILC2s were sorted separately as biological replicates from the whole lungs of 3 C57Bl/6J female mice and 3 CC030 female mice. Single cell suspensions from lungs were

enriched for ILC2 with the Lineage Cell Depletion kit, mouse (Miltenyi™) and the CD25 MicroBead Kit, mouse (Miltenyi™), utilizing LD columns (Miltenyi) for both separations. ILC2 for this experiment were identified by surface staining as Lymphocytes (FSC-A vs. SSC-A), singlets by pulse gating, Live/Dead Aqua-, Lineage- (α-CD3, α-CD4, α-FCεR1, Hematopoietic Lineage Labeling Cocktail, anti-mouse biotin (Miltenyi™), CD90+, CD127+, CD25+, ICOS+. Sorting was performed on a BD FACS Aria III run on the slowest setting with a 100µM nozzle. Due to equipment availability changes, ILC2 sorted for assessment of Ffar3 expression (Figure 5) and culture (Figure 4, 6, 7) were sorted on a BD FACS Symphony S6 and defined as Lymphocytes (FSC-A vs. SSC-A), singlets by pulse gating, Live/Dead Aqua-, Lineage- (α-CD3, α-CD4, α-FCεR1, Hematopoietic Lineage Labeling Cocktail, anti-mouse biotin (Miltenyi™), CD90+, CD127+, ST2+. Cells were again sorted on the lowest setting with a 100µM nozzle. Analytical flow cytometry of cultured ILC2s: All ILC2 harvested from culture were stained for viability, and human ILC2 were underwent surface staining for EGFR (antibody) for 15 minutes prior to fixation. Cells were fixed with the FoxP3 Transcription Factor Staining Buffer Kit (eBioscience™) kit. Intracellular cytokine staining and Ki-67 staining was carried out for 45 minutes, and cells were not restimulated prior to intracellular cytokine staining. Mouse EGFR staining was performed by incubating cells with concentration of the unconjugated α-EGFR antibody (Invitrogen,) for 30 minutes. Cells were washed and subsequently incubated with a 1:1,000,000 dilution of the secondary antibody for an additional 30 minutes. 123count eBeads™ (Invitrogen) were utilized to enumerate ILC2. Cultured cells were analyzed on a 4-laser Cytek® Aurora spectral flow cytometer. Human ILC2 sorting: Phlebotomy was performed on healthy, non-asthmatic participants to recover 50mL of blood from each donor. PBMCs were isolated using SepMate™ tubes with Lymphoprep™ (StemCell). Red blood cells lysis was carried out with ammonium chloride-based RBC Lysis Buffer (Tonbo). Lineage negative cells were enriched with the Lineage Cell Depletion Kit, human (Miltenyi) and stained for viability with the LIVE/DEAD™ Fixable Aqua Dead Cell Stain Kit (Invitrogen) in PBS. Surface staining for ILC2 was carried out for 20 minutes in 3% FBS with anti-human lineage cocktail (Biolegend), α-CD45, α-CD127, and α-CRTH2. ILC2 were sorted on a BD FACS Symphony™ S6 cell sorter as Lineage-, CD45+, CD127+, CRTH2+.

Instrument	Analystical: BD 5-Laser Fortessa, 4-laser Cytek® Aurora. Sorting: BD FACS Aria III, BD FACS Symphony S6
Software	FlowJo (v10.8.1)
Cell population abundance	Cell population abundance was determined via count beads (123count eBeads). A known quantity of beads was added to a sample, and the ratio of expected bead to counted beads was used to determine the total numbers of a cell population in the analyzed sample. The volume taken for each flow cytometry sample was recorded, and so the total number of cells in the original samples (i.e. whole lung) could be calculated.
Gating strategy	The gating strategies for all relevant cell populations have been shown in the figures and supplement of the manuscript.

☒ Tick this box to confirm that a figure exemplifying the gating strategy is provided in the Supplementary Information.
